# Concentration Effects in Peptide–Lipid Bilayer Interactions: A Coarse-Grained Molecular Dynamics Study

**DOI:** 10.1021/acs.jpcb.5c07780

**Published:** 2026-03-16

**Authors:** Aleksandra Drajkowska, Andrzej Molski

**Affiliations:** 467899 Adam Mickiewicz University in Poznań, Faculty of Chemistry, ul. Uniwersytetu Poznańskiego 8, 61-614 Poznań, Poland

## Abstract

In this work, we explore the assumption and predictions of a two-state model by Huang and collaborators that was developed to quantify peptide-induced pore formations in lipid bilayers. Specifically, we report on Martini 2 and Martini 3 molecular dynamics simulations of melittin, a bee venom-derived peptide with antimicrobial properties, and an amyloid peptide fragment Aβ(29–42) interacting with a POPC bilayer. We focus on the effects of peptide concentration on the cooperative peptide–lipid bilayer interactions. We found that peptides first adsorb at the membrane interface and then, beyond a critical peptide-to-lipid ratio, insert into the membrane to form transmembrane clusters. However, in contrast to Huang’s model, this cooperative effect does not coincide with a turnover in the rate of membrane thinning. This difference may stem from the peptide concentration dependence of the bilayer mechanical properties that is not accounted for in Huang’s model. We also found that antimicrobial melittin and amyloidogenic Aβ(29–42) show similar characteristics of peptide–lipid interaction. This supports the conjecture that antimicrobial peptides and amyloidogenic peptides may use the same molecular mechanism to disrupt lipid membranes.

## Graphical abstract

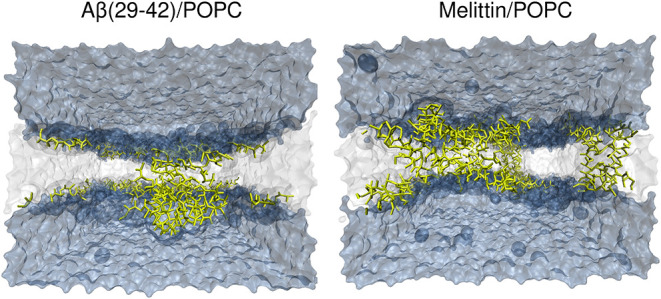

## 1 Introduction

The physical chemistry of peptide-lipid interactions is a subject of continuous research interest.^1–3^ Both antimicrobial and amyloid peptides can affect lipid bilayers.^4,5^ Antimicrobial peptides can compromise the integrity of microbial membranes, thereby preventing the growth of bacteria, fungi, or parasites.^6,7^ Amyloid peptides are closely associated with the pathogenesis of neurodegenerative diseases, as they can form toxic aggregates that disrupt lipid membranes and impair cellular function.^8–11^ The similarities between these two classes of peptides suggest potential links between their mechanisms of action.

Several quantitative models for the peptide-membrane interaction have been put forward to explain the peptide adsorption, insertion, and self-organization.^12–16^ Because of the complexity of the peptide-lipid interactions, the validity ranges of these model are not well established. Here we explore a two-state model put forward by Huang and co-workers^14,15^ that describes the concentration effects in peptide-lipid bilayer interactions. In this model, peptides first adsorb on the bilayer surface (state S) in a parallel orientation, and then, above a critical peptide-to-lipid ratio (*P*/*L*)*, insert into the membrane (state I) with a perpendicular orientation. Huang’s model was quantified through a free-energy approach,^17–20^ which assumes that membrane thinning is associated with surface area extension creating internal membrane tension. Experimental studies using X-ray and neutron diffraction showed that the membrane thickness, *h*, decreases linearly with increasing *P*/*L* up to (*P*/*L*)*, and then levels off. The model predicts that the area per lipid, *A*_L_, increases with peptide concentration up to (*P*/*L*)* and then levels off, and that the fraction of inserted peptides, ϕ, changes linearly with the inverse peptide-to-lipid ratio, 1/(*P*/*L*).

In this work we explore the assumption and predictions of Huang’s two state-model through coarse-grained molecular dynamics simulations of two peptides interacting with POPC bilayer: amyloid peptide fragment Aβ(29–42) and melittin, a bee venom-derived peptide with antimicrobial properties. Interactions of melittin with lipid peptides have been studied both experimentally and through molecular dynamics simulations.^21–23^ The melittin-POPC interaction parameters for Huang’s free energy model of system are available in^15^ which allows a direct comparison with simulations. Amyloid peptide fragments are often used as simplified models of the full-length Aβ amyloidogenicity and toxicity.^24–27^ We chose Aβ(29–42) for this study to explore the conjecture that antimicrobial and amyloid peptides share common mechanisms of lipid-peptide interactions.^4^ Also we wanted to compare the effect of a short peptide, Aβ(29–42), that cannot span a lipid membrane with a longer one, melittin, that can span the membrane. Despite their different physicochemical properties, with Aβ(29–42) being predominantly hydrophobic and melittin having a positively charged C-terminus, both peptides are amphipathic and known to interact with lipid membranes.

Molecular dynamic simulations can reveal molecular aspects of peptide-lipid interactions.^28^ In this work, we use the Martini 2.2 coarse-grained force field^29^ and, for comparison, the Martini 3.0 force field.^30^ An advantage of coarse-grained over all-atom simulation is that one can study larger system for longer times.^31^ The Martini 2.2 force field has been successfully used to study peptide-lipid interactions,^32^ including amyloid peptides.^33–36^ The Martini 3.0 model is a refined version of Martini 2.2, developed to amend the limitations of Martini 2.2.

We found that our Martini 2.2 simulations show strong Aβ(29–42) and melittin interactions with POPC, leading to peptide adsorption and membrane insertion. In accord with Huang’s two-state model, at low peptide concentrations the peptides adsorb parallel to the membrane surface but do not insert into the bilayer. The peptide adsorption causes membrane thinning. Above a threshold concentration, peptides insert cooperatively into the membrane and form transmembrane clusters. The fraction of inserted peptides changes (roughly) linearly with the inverse of the peptide-to-lipid ratio. However, our Martini 2.2 simulations are only partly consistent with Huang’s two-state model. For instance, in contrast with Huang’s model, the peptide threshold concentration, (*P*/*L*)*, is significantly lower than that when the membrane thickness levels off.

In our simulations, the Martini 3.0 force field showed weaker lipid-peptide interactions than Martini 2.2. For melittin interacting with POPC, we observed only partial peptide adsorption. For Aβ(29–42), we found stronger adsorption but still no peptide insertions into the membrane. In our case, Martini 2.2 seems to better represent the peptide-lipid interactions that can lead to the loss of membrane integrity.

The paper is structured as follows. The next section describes our simulation setup and data analysis methods. The Results section is divided into the presentation of the Martini 2.2 simulations and that for Martini 3.0. The Discussion section attempts to rationalize three issues: the partial consistency of Huang’s model and our simulations, the similarity of Aβ(29–42) and melittin mechanisms of action, and the discrepancies between the Martini 2.2 and Martini 3.0 simulations of peptide-lipid interactions. Finally, we present the conclusions of our work.

## 2 Methods

### 2.1 Simulation Setup

In this work we focus on the interactions of two peptides, amyloid peptide fragment Aβ(29–42) (sequence: GAIIGLMVGGVVIA) and antimicrobial peptide melittin (sequence: GIGAVLKVLTTGLPALISWIKRKRQQ), with palmitoylolyoilphosphatidylcholine (POPC) lipid bilayer. The initial atomistic structure of melittin was taken from an X-ray structure (PDB: 2MLT).^37^ For Aβ(29–42), the structure was extracted from the C-terminal region of a NMR structure of monomeric Aβ(1–42) (PDB: 1IYT).^38^ The atomistic structures were converted to Martini coarse-grained representations using martinize.py (Martini 2.2) and Martinize2 (Martini 3.0).^39,40^ The secondary structure was assigned using DSSP and used only to define the coarse-grained mapping. No elastic network or other additional structural restraints were applied. For the Martini 3.0 mapping, we disabled the side chain corrections, since this feature led to instability in our melittin simulations.

The simulated systems contained bilayer POPC lipids, Aβ(29–42) or mellitin peptides, Na^+^ and Cl^–^ ions, and water W beads. The Na^+^ and Cl^–^ ions were used to neutralize the systems and create a 0.15 mM NaCl solution. The Aβ(29–42) and POPC systems contained 512 lipids and 4–74 peptides, whereas the melittin and POPC systems comprised 512 lipids and 2–32 peptides, depending on the simulation. Further system-specific details are provided in Tables S1 and S2 in Supporting Information.

To overcome potential kinetic traps in peptide-lipid interactions, we applied a system self-assembly protocol, where the simulations start with a random mixture of peptides, lipids, and solvent.^29^ The initial configuration was prepared by randomly placing lipids, peptides, and solvent beads in the simulation box. Both lipids and peptides were initially placed in the same slab centered at *z* = 0 with dimensions *l* × *l* × *l*/2, where *l* = 15 nm is the cubic simulation box edge length, to facilitate the spontaneous self-assembly of the bilayer in the *xy* plane. This protocol does not simulate the kinetic process of peptide insertion into a preformed membrane. Instead, it is intended to help reach the thermodynamic equilibrium where peptides may partition between the aqueous phase, membrane surface, and membrane interior according to their thermodynamic preferences. This protocol also mimics Huang’s equilibrium conditions where the peptide-membrane systems were prepared from homogeneous mixtures.

In all simulations we used the *NPT* ensemble at 1 atm and 310 K. Temperature was controlled with the velocity-rescale thermostat. For pressure, we used the Berendsen barostat with isotropic coupling during the self-assembly stage and switched to the Parrinello–Rahman barostat with semi-isotropic coupling for production runs. The integration time step was 0.02 ps. A cutoff distance of 1.1 nm was applied for nonbonded van der Waals interactions. Most molecular dynamics parameters were kept at their default values. However, during the self-assembly and production stages, the LINCS algorithm was used for enforcing bond constraints, with the order of expansion set to 6 and the number of iterations increased to 2 to improve stability. Bond constraints were applied because initial test simulations of the melittin system without constraints showed instability, with the system becoming unstable in both force fields. To ensure consistent simulation conditions across all systems and enable direct comparison between different peptides and force field versions, bond constraints were applied uniformly to all simulations. Since all our simulations were performed under identical constraint conditions, the relative trends and comparisons between systems should remain valid.

In Martini 2.2 simulations, the initial system self-assembly was run for 5 μs and then the production was set to 10 μs. We noticed an increased data scattering at high peptide concentrations, so we ran three repeats for each simulation setup to reduce the statistical noise.

For the Martini 3.0 systems, the bilayer self-assembly was much faster than for Martini 2.2, so we limited the self-assembly runs to 200 ns. The Martini 3.0 production runs were set to 15 μs and were not repeated, as we found consistently only weak peptide-lipid interactions within this model.

The force field files, simulations files, and starting/final configurations for our simulations are available at https://github.com/adrajkowska/peptide-lipid-bilayer-simulations.

### 2.2 Data Analysis

Our simulations attempt to quantify the effect of peptide surface adsorption and insertion on the membrane properties. We determine the membrane thickness, *h*, the area per lipid *A*_L_, the compressibility modulus *K*_a_, and the fraction of inserted peptides, ϕ.

The membrane thickness, *h*, is defined here as the average distance between the PO4 beads in the upper and lower leaflets. We used the GROMACS distance tool to compute the time-dependent center-of-mass distance between the PO4 beads and the average distance is reported as *h*.

The area per lipid, *A*_L_, and the membrane compressibility modulus, *K*_a_, were obtained from the time trace of surface area fluctuations using the GROMACS energy tool and an in-house python script. The area per lipid, *A*_L_, was calculated as the ratio of the average *xy*-area of the simulation box, ⟨*A*_*xy*_⟩, and the number lipids per leaflet, *L*/2: *A*_L_ = ⟨*A*_*xy*_⟩/(*L*/2). The area compressibility modulus was determined as

1 $$K_{a}=\frac{k_{B}T\left\langle A_{\mathrm{xy}}\right\rangle }{\left\langle {\left(\delta A_{\mathrm{xy}}\right)}^{2}\right\rangle }$$

where ⟨(*δ A*_*xy*_)_2_⟩is the mean square fluctuation of *A*_*xy*_, *k*_B_ is Boltzmann’s constant, and *T* is the temperature.

The fraction of inserted peptides, ϕ, was determined using an in-house Python script that counts the peptides whose center of mass lies within the membrane core. The final value of ϕ is calculated as the number of inserted peptides divided by the total number of peptides in the system. According to Huang’s theory, the inserted peptide fraction, ϕ, changes with the peptide-to-lipid ratio, *P*/*L*, as

2 $$\phi =\frac{1}{1-\beta }\left[1-\frac{{\left(P/L\right)}^{*}}{\left(P/L\right)}\right]$$

which can be visualized as linear plot of ϕ against 1/(*P*/*L*) up to 1/(*P*/*L*) *. The slope is determined by the dimensionless β that can be compared with the experimental value. In our simulations, the relation ϕ vs *P*/*L* is not clearly visible due to the statistical noise.

The numerical values for representative properties of the simulated systems are given in Tables S3 through S6 in Supporting Information.

## 3 Results

In this section we assess the assumptions and predictions of Huang’s two-state model that can be directly tested in MD simulations. We show how the membrane thickness, *h*, the area per lipid, *A*_L_, and the fraction of inserted peptides, ϕ, change as functions of the *P*/*L* ratio. Moreover, we show that the membrane volume per lipid, *V*_L_ = *h* × *A*_L_, and the compressibility modulus, *K*_a_, change with *P*/*L*, which is not accounted for in Huang’s model. We begin our presentation with the Martini 2.2 simulations followed by the results for Martini 3.0.

### 3.1 Martini 2 Simulations

In the experiments of Huang et al., the membrane thickness decrement, Δ*h*, changes linearly with the *P*/*L* ratio. At high peptide concentrations, (*P*/*L*) > (*P*/*L*)*, the thickness, *h*, level off. This was interpreted as an indication that an effective insertion of the peptides into the membrane begins at (*P*/*L*) *.

Figures 1 and 2 show how the membrane thickness, *h*, and the area per lipid, *A*_L_, change with the *P*/*L* ratio for Aβ(29–42) and melittin. The simulations show that for Aβ(29–42), a minimum (threshold) peptide concentration is needed for the formation of transmembrane clusters, whereas for melittin, transmembrane clusters form already at the lowest tested concentration. This suggests that the transmembrane clustering is a cooperative effect, particularly pronounced for the shorter Aβ(29–42) peptide. A visual inspection of the simulation trajectories shows also, that below the concentration threshold (black circles), the peptides lie flat on the membrane surface, whereas at high peptide concentrations (red circles), some of the peptides assume a perpendicular orientation, which is consistent with Huang’s model (see Figure 3). The perpendicular peptide orientation is characteristic of transmembrane clusters. In our simulations, we observe that at high peptide concentrations, peptides organize into transmembrane clusters, whereas at low concentrations they remain parallel to the membrane surface.

**Figure 1:**
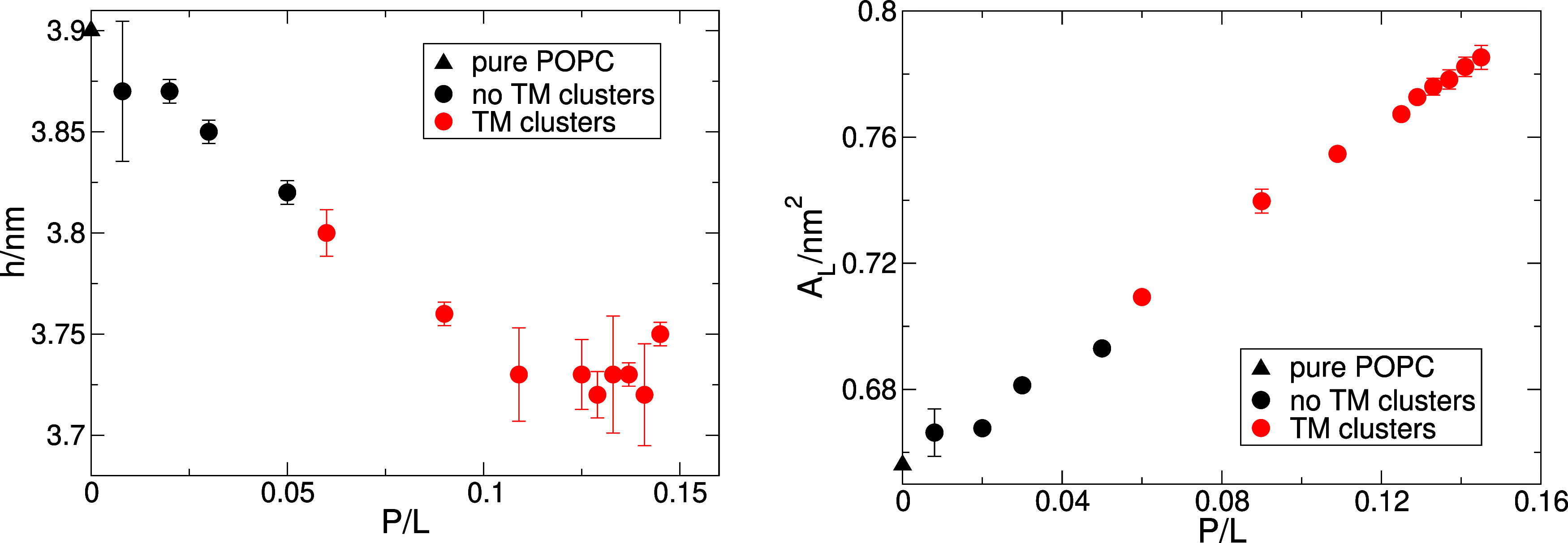
Aβ­(29–42)/POPC, Martini 2.2: the membrane thickness, *h*, and area per lipid, *A*_L_, as functions of the peptide-lipid ratio, *P*/*L*, in Martini 2.2 simulations of Aβ(29–42)/POPC systems. The black triangle indicates pure POPC membrane, black circles indicate the simulations with no transmembrane clusters, whereas the red circles indicate the presence of transmembrane clusters.

**Figure 2:**
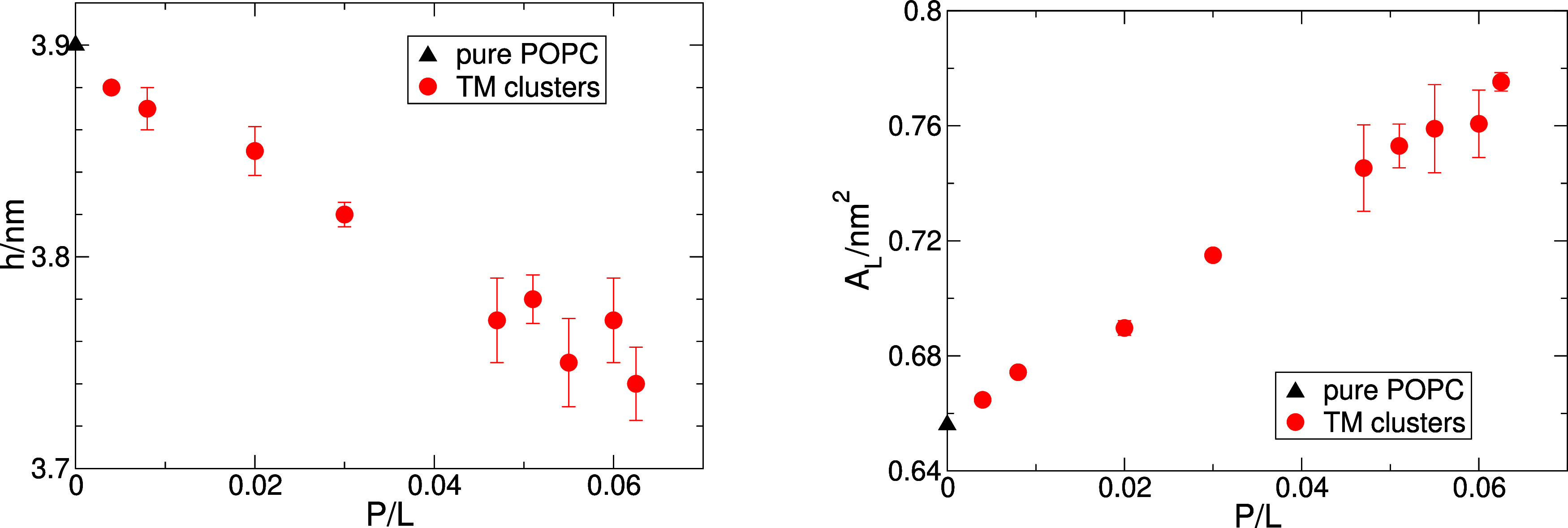
Melittin/POPC, Martini 2.2: the membrane thickness, *h*, and area per lipid, *A*_L_, as functions of the peptide-lipid ratio, *P*/*L*, in Martini 2.2 simulations of melittin/POPC systems. The black triangle indicates pure POPC membrane, whereas the red circles indicate the presence of transmembrane clusters.

**Figure 3:**
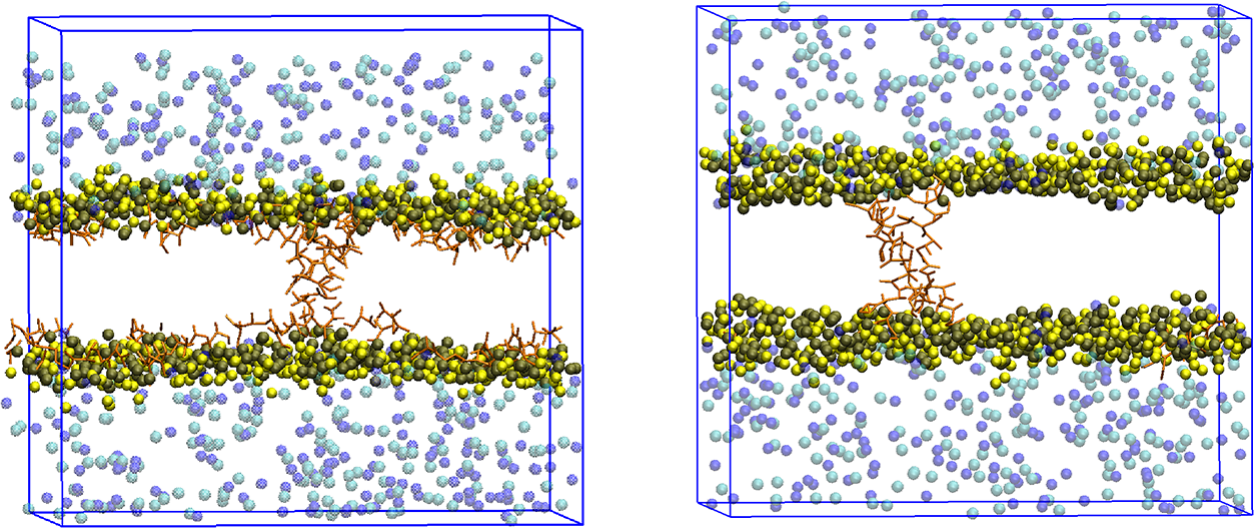
Snapshots from Martini 2.2 simulations showing peptide orientations at high peptide concentrations. Left panel: Aβ(29–42)/POPC system showing peptides adsorbed at the membrane surface and peptides inserted in the membrane. Right panel: melittin/POPC system showing inserted melittin peptides in perpendicular transmembrane orientation. Peptides are shown in orange, PO4 beads in yellow, NC3 beads in moss green and ions in light and dark blue.

It is instructive to compare the threshold ratio (*P*/*L*)* for transmembrane clustering with the *P*/*L* ranges where the thickness, *h*, levels off. For Aβ(29–42) in Figure 1, (*P*/*L*)* ≈ 0.053, whereas *h* begins to level off between *P*/*L* = 0.111 and 0.145, stabilizing at values around 3.72 to 3.75 nm. Figure 2 shows that for mellitin (*P*/*L*)* ≤ 0.004, whereas *h* levels off only at *P*/*L* between 0.047 and 0.063, where it reaches values from 3.74 to 3.77 nm. Note that the area per lipid, *A*_L_, expands linearly in the entire peptide concentration range for both Aβ(29–42) and melittin.

Clearly, melittin shows a lower *P*/*L* threshold for transmembrane clustering than Aβ(29–42), reflecting a higher propensity for transmembrane clustering. Nevertheless, the *P*/*L*-dependences for melittin and Aβ(29–42) are qualitatively similar. Above a threshold peptide concentration, (*P*/*L*)*, both the membrane thickness, *h*, and the membrane area per lipid, *A*_L_, continue to change linearly with no apparent change in the slope. Within the studied peptide concentration range, the membrane thickness, *h*, begins to level off only at the highest peptide concentrations, while the area per lipid, *A*_L_, continues to increase. This behavior of *h* and *A*_L_ for both melittin and Aβ(29–42) deviates from the predictions of Huang’s model.

Figure 4 shows the fraction, ϕ, of inserted Aβ(29–42) and melittin peptides plotted against the inverse peptide-to-lipid ratio, 1/(*P*/*L*), for the peptide-lipid ratios lower than 1/(*P*/*L*)*. The simulated points are plotted against the fitted straight lines and indicate an approximate consistency with Huang’s model predicting linear plots. For Aβ(29–42), the fraction of inserted peptides remains zero at low concentrations (*P*/*L* = 0.008 to 0.047), indicating that this short peptide initially only adsorbs on the membrane surface without penetrating the bilayer core. Insertion begins at *P*/*L* = 0.063 with ϕ = 0.19, and increases to ϕ = 0.77 at *P*/*L* = 0.093, demonstrating a sharp transition from surface adsorption to membrane insertion. The fraction then fluctuates between 0.28 and 0.59 at higher concentrations, suggesting dynamic equilibrium between the inserted and surface-bound peptides.

**Figure 4:**
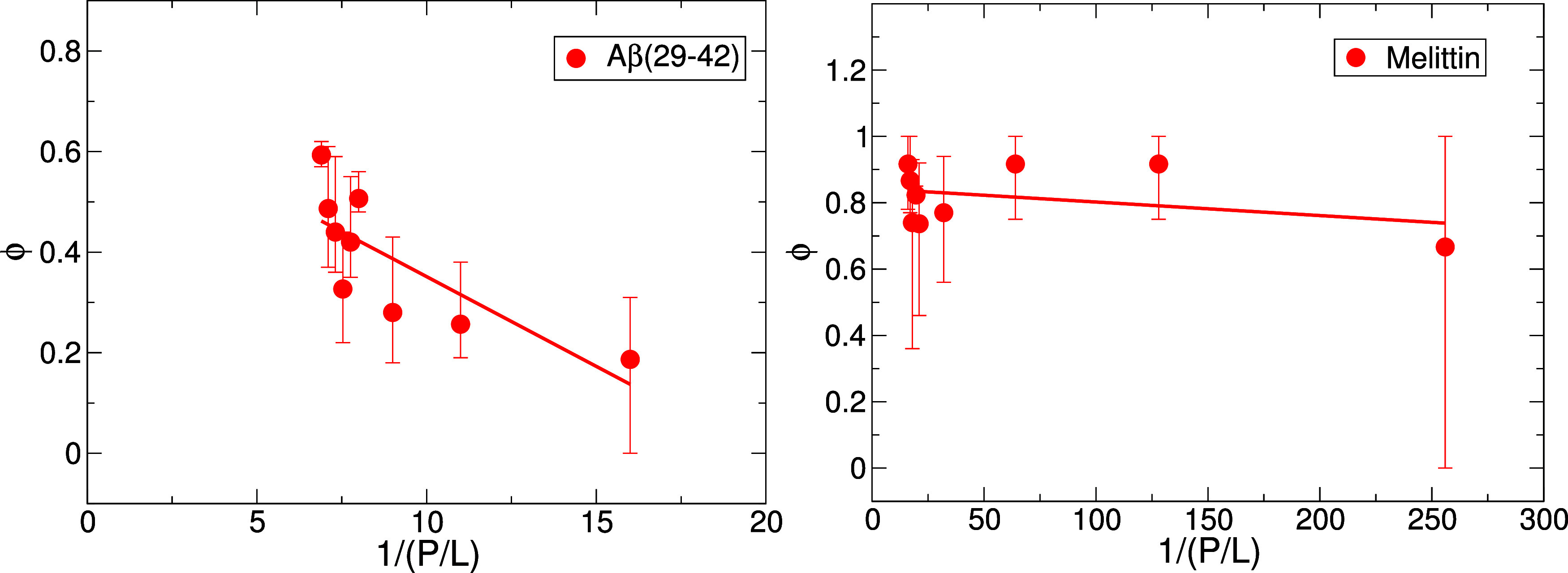
Fraction of inserted peptides, ϕ, as a function of the peptide-lipid ratio, *P*/*L*, in Martini 2.2 simulations of Aβ(29–42) and melittin/POPC systems. The straight lines indicate linear fits. Note the large scattering of data points.

In contrast, melittin shows stronger insertion capability, with ϕ = 0.67 already at the lowest tested concentration, *P*/*L* = 0.004. The insertion fraction increases to ϕ = 0.92 at *P*/*L* = 0.008 and remains consistently high (0.74 to 0.92) across all tested concentrations. This behavior correlates with melittin’s length that is sufficient to span the membrane, which supports transmembrane penetration even at very low concentrations. As Aβ(29–42) is too short to span the membrane, several molecules need to act cooperatively to form a transmembrane cluster, which increases (*P*/*L*)*.

Huang’s two-state model links the membrane thinning, Δ*h*, with the surface area expansion, Δ*A*, that creates membrane tension. Assuming that the hydrocarbon tails behave as an incompressible liquid one gets Δ*A* /*A*_0_ ≈ −Δ*h*/*h*_0_. Figure 5 shows, however, that this assumption is not valid in our simulations, as the increase in the membrane area exceeds the decrease in the thickness. Despite the structural and functional differences between the peptides, both exhibit similar trends. This suggests a general mechanism by which amphipathic peptides interact with lipid bilayers, promoting surface expansion while moderately reducing thickness. Such behavior may represent a common physicochemical feature of peptide-lipid interactions rather than being peptide-specific.

**Figure 5:**
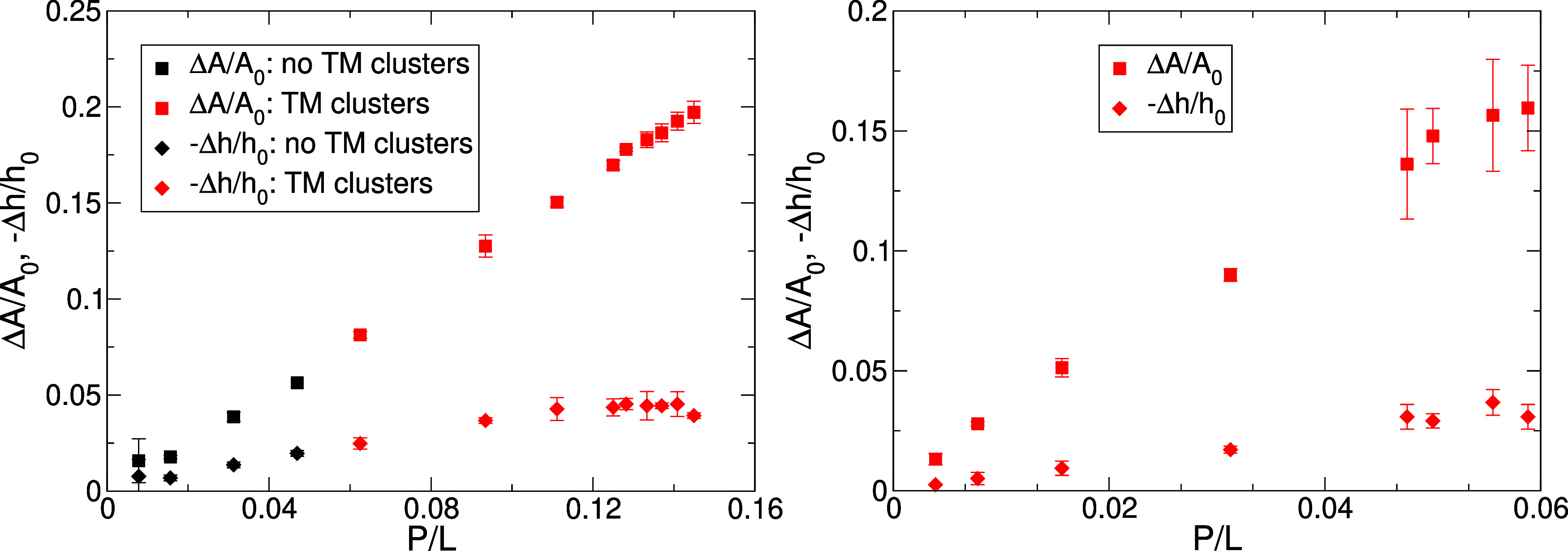
Fractional area expansion, Δ*A*/*A*_0_, and fractional thinning, Δ*h*/*h*_0_, as a function of the peptide-lipid ratio, *P*/*L*, for Aβ(29–42)/POPC (left panel) and melittin/POPC (right panel) systems in Martini 2.2 simulations.

Figure 6 shows the peptide-to-lipid ratio, *P*/*L*, dependence of the area compressibility modulus, *K*_a_, for Aβ(29–42)/POPC (left panel) and melittin/POPC (right panel). The parameter *K*_a_ measures membrane resistance to surface stretching, with higher values indicating greater stiffness. For both peptides, *K*_a_ decreases progressively with increasing *P*/*L*, signaling membrane softening and reduced mechanical stability. Specifically, *K*_a_ declines from approximately 260 to 190 pN/nm for Aβ(29–42) and from about 250 to 160 pN/nm for melittin. Although the melittin system exhibits a slightly larger decrease, the overall reductions are comparable. This similarity supports the hypothesis of a shared physicochemical mechanism whereby peptide binding enhances membrane elasticity and diminishes structural integrity.

**Figure 6:**
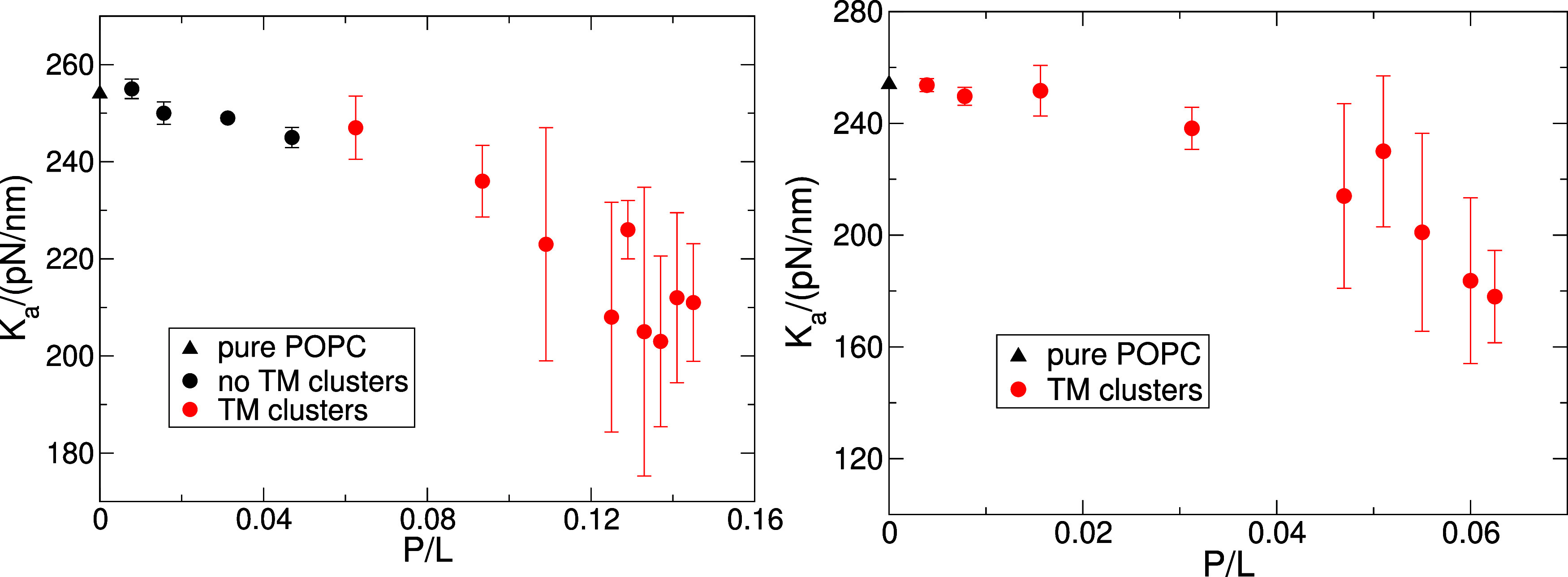
Area compressibility modulus, *K*_a_, as a function of the peptide-to-lipid ratio, *P*/*L*, for Aβ(29–42)/POPC (left panel) and melittin/POPC (right panel) systems in Martini 2.2 simulations.

### 3.2 Martini 3.0 Simulations

The Martini 3.0 simulations revealed different behavior compared to Martini 2.2 for both Aβ(29–42) and melittin systems.

For the Aβ(29–42)/POPC system, the membrane thickness, *h*, decreases continuously without showing the characteristic leveling off observed in Martini 2.2 simulations, see Figure 7. The area per lipid, *A*_L_, exhibits similar trends to those observed in Martini 2.2, suggesting that the force field changes primarily affect the membrane thinning mechanism rather than the surface area expansion. The compressibility modulus, *K*_a_, for Aβ(29–42) in Martini 3.0 shows a more pronounced decrease compared to Martini 2.2, dropping from approximately 208 to 140 pN/nm over the tested concentration range, see Figure 7. This suggests that while the membrane becomes more flexible in Martini 3.0, the underlying mechanism differs from that observed in Martini 2.2, where the decrease was associated with peptide insertion and transmembrane cluster formation. The biggest difference was observed in the peptide insertion behavior. In Martini 3.0 simulations, the fraction of inserted peptides, ϕ, remained zero across all tested concentrations for Aβ(29–42), indicating that this peptide does not penetrate the membrane core in the Martini 3.0 force field. This contrasts with Martini 2.2, where peptide insertion was observed above the threshold concentration.

**Figure 7:**
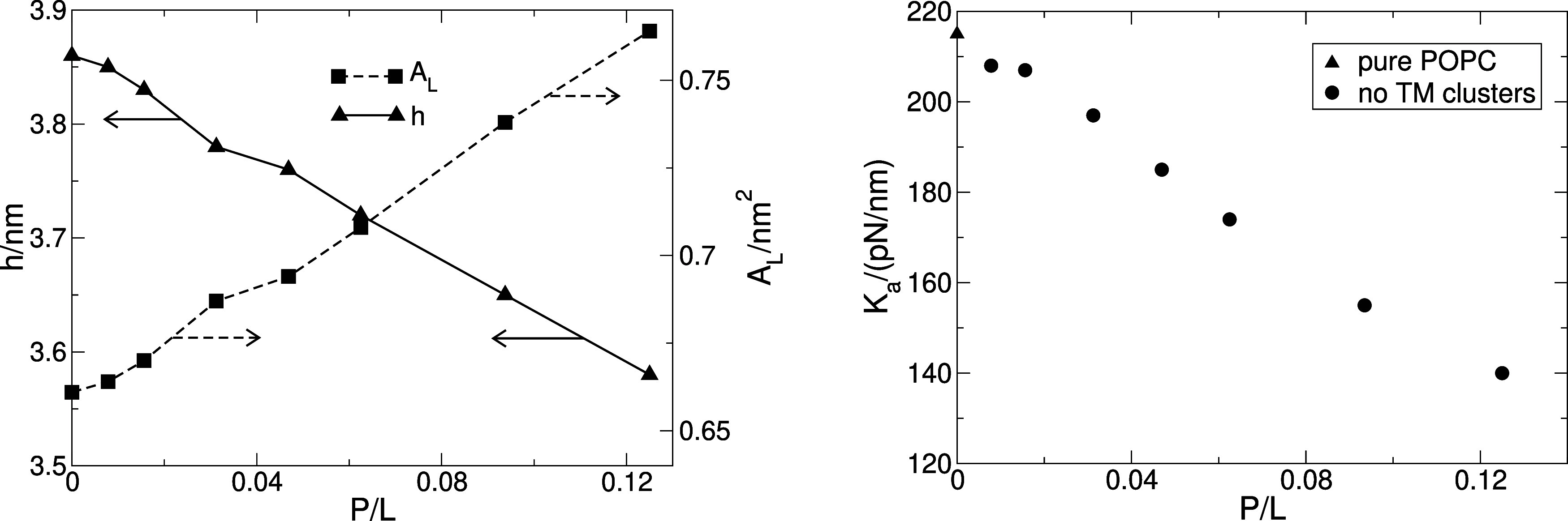
Aβ­(29–42)/POPC, Martini 3.0: mechanical properties of POPC bilayer interacting with Aβ(29–42) peptides, simulated with the Martini 3.0 force field. Left panel: Membrane thickness, *h*, and area per lipid, *A*_L_, as functions the peptide-to-lipid ratio, *P*/*L*. Right panel: Area compressibility modulus, *K*_a_, as a function of *P*/*L*.

For melittin/POPC systems in Martini 3.0, the peptides showed only minimal surface adsorption and did not form transmembrane clusters. The membrane properties did not change significantly with increasing peptide concentration and did not exhibit clear trends or characteristic patterns, as evidenced by the lack of significant changes in membrane thickness and area per lipid. For melittin/POPC systems in Martini 3.0, the compressibility modulus remains relatively constant around 150–200 pN/nm, showing minimal variation with peptide concentration. This behavior is consistent with the lack of significant peptide-membrane interactions observed in these simulations.

## 4 Discussion

### 4.1 Huang’s Two-State Model is Only Partly Consistent with the Simulations

As illustrated in Table 1, our coarse grained simulations showed that Martini 2.2 reproduces semiquantitatively some elasticity properties of pure POPC and POPC interacting with melittin. This is an expected outcome as Martini 2.2 is a successful model to mimic the membrane lipid interactions.

**Table 1:** Comparison of the Simulated and Experimental Properties of Pure POPC Membrane and POPC Membrane with Melittin and Aβ(29–42)^c^

	Martini 2.2	Experiments
Pure POPC		
*K*_a_ (pN/nm)	254	240^a^
*h* (nm)	3.90	2.75^b^
*A*_L_ (nm^2^)	0.66	0.68

POPC with	Aβ(29–42)	Melittin	Melittin
*A*_P_ (nm^2^)	0.75	2.25	2.23
(P/L)^a^	0.053	0.004	1/62
β	–0.036	–0.0004	–0.39

a In Huang’s free energy model, *K*_a_ is treated as a constant parameter, whereas in our simulations *K*_a_ decreases with peptide concentration.

b The quoted experimental thickness value is the phosphate-to-phosphate distance reduced by 1 nm, *h* – 1 nm, whereas our simulation *h* is defined as the distance between PO4 beads.

c Only simulation data are available for Aβ(29–42). The table presents the area compressibility modulus, *K*_a_, membrane thickness, *h*, area per lipid, *A*_L_, area expansion per peptide, *A*_p_, threshold peptide-to-lipid ratio (*P*/*L*)*, and the β parameter describing the effect of pores on membrane thickness parameter. Our simulation values are shown alongside experimental data for POPC and melittin reproduced from ref 19. Copyright 2004 American Chemical Society.

Our Martini 2.2 simulations are partly consistent with Huang’s model. The similarities include the initial linear decrease in membrane thickness with increasing peptide concentration, the formation of transmembrane structures above a threshold concentration, the cooperative nature of peptide insertion, and the overall trend of membrane thinning upon peptide binding.

However, as demonstrated in the previous section our Martini 2.2 simulations reveal important deviations from Huang’s model. In Huang’s experiments, the membrane thickness was reported to level off above the threshold concentration, which was interpreted as evidence that peptide insertion relieves the membrane tension created by surface adsorption. However, our simulations show that, while the thickness does eventually level off at high peptide concentrations, this turn over occurs at a significantly higher *P*/*L* ratio than the threshold for transmembrane cluster formation. Moreover, the area per lipid continues to increase even when the thickness stabilizes. This indicates that the membrane continues to expand despite the saturation in thickness reduction.

These discrepancies may arise from several factors. First, we note the different experimental and simulation setups. In our simulations we follow peptide-lipid interactions in free, equilibrated lipid-membrane, whereas in Huang’s experiments focus on layered membrane samples. This may not be an issue, as Huang’s experiments were performed for well hydrated lipid bilayers that suggests independent behavior of individual layers.

Second, Huang’s model was developed for equilibrium system, whereas the simulation equilibration time is a challenge for membrane system. We tried to minimize this issue by applying a self-assembly protocol where we do not impose the initial peptide-bilayer asymmetry. We note, that many peptide-membrane experiments and simulation place peptides at one side of the membrane. This asymmetric arrangement corresponds to the actual mode of action of membrane active membranes. On the other hand, an asymmetric simulation protocol probes the kinetic snapshots of the interactions and may not reach an equilibrium state.

Finally, the limited system size of our simulations may affect the equilibrium averaging of the results. For instance, the membrane thickness, *h*, is determined here as the average distance between the PO4 beads in both leaflets. This assumes that the membrane thickness is uniform across the membrane. However, the presence of the surface and trans-membrane clusters makes the membrane nonuniform in our simulations. Such effects are not allowed for in Huang’s free energy model and may be the reason why this model is only partly consistent with the simulations.

### 4.2 Aβ­(29–42) and Melittin Show Similar Mechanisms of Action

Our simulations show clear quantitative differences between peptide-lipid interaction for melittin and Aβ(29–42). An important distinction between the two peptides is their propensity for cluster formation. Melittin forms transmembrane clusters already at *P*/*L* = 0.004, while Aβ(29–42) requires a higher concentration of *P*/*L* = 0.063 to initiate cluster formation. This difference likely reflects the longer length of melittin (26 residues) compared to Aβ(29–42) (14 residues), which may facilitate transmembrane orientation and cluster formation. The easier cluster formation for melittin may contribute to the earlier onset of thickness flattening observed in the melittin system. Dispite of these differences, our simulations suggests that both may disrupt membranes through a common mechanism of action, i.e., through the formation of transmembrane clusters.

The mechanism of action of membrane active peptides depends on both the peptide chemical structure and the membrane lipid composition. Two broad mechanisms have been proposed to explain peptide-induced membrane disruption. In the carpet mechanism, peptides adsorb on the surface of a membrane, which, at high peptide concentration, leads to disruptive membrane thinning or lipid micellization. The carpet mechanism is sometimes linked with peptides that are too short to span the cell membrane.^41^ For instance, the short 13-residue aurein 1.2 peptide was found to disrupt membranes via the carpet mechanism.^42^ However, it was found in another study that the membrane lipid composition may support the aurin pore stability.^43^ Moreover, the carpet mechanism for aurin was challenged in.^44^ In the pore formation mechanism, peptides form membrane-spanning pores that compromise the membrane’s integrity. For instance, melittin can span the membrane and is believed to disrupt membranes via pore formation.^3^ However, as with aurin and other peptides, the melittin mechanism of action is not entiertly clear, as the carpet mechanism was also postulated.^45,46^

In our simulations, both Aβ(29–42) and melittin can form transmembrane clusters. However, different lengths of Aβ(29–42) and melittin lead to different peptide arrangements. Aβ(29–42) cannot span the full bilayer thickness, but instead forms transmembrane clusters through stacking arrangements where peptides align on top of each other. In contrast, melittin can span the entire membrane and forms ordered transmembrane structures. These differences in the cluster architecture arise primarily from the different peptide lengths rather than from belonging to different classes of peptides. This observation supports the hypothesis that both antimicrobial and amyloid peptides may share common fundamental mechanisms of membrane disruption, with structural differences primarily affecting the specific arrangement of peptides within the membrane rather than the overall mode of action.

Martini force field has inherent limitations in representing detailed β-sheet structures. The model uses isotropic beads that lack directionality, which is essential for capturing hydrogen bonds and protein folding.^32^ In Martini, backbone residues are typically represented by single beads,^29^ and hydrogen bonds are not explicitly modeled. The secondary structure is maintained through backbone dihedral angle potentials, which preserve the input secondary structure but prevent dynamic changes or spontaneous formation of new β-sheet stacks during simulation.^29^ Therefore, while the stacking arrangements observed in our simulations may reflect peptide–peptide interactions that could lead to β-sheet formation, the coarse-grained model cannot directly confirm whether these structures represent true β-sheet stacks as would be defined at atomistic resolution. To our knowledge, direct experimental evidence for Aβ(29–42) transmembrane clusters in lipid bilayers is currently lacking, and further experimental work would be needed to confirm whether such clusters form under physiological conditions.

Our findings on melittin transmembrane cluster formation are consistent with the recent molecular dynamics studies of antimicrobial peptides.^23^ Sun et al. reported that melittin peptides adopt either transmembrane (T-peptide) or U-shaped conformations after spontaneous penetration into lipid bilayers, with both conformations aggregating to form stable pores.^23^ The spontaneous penetration was observed when the peptide-to-lipid ratio exceeded approximately 2/100 in DPPC/POPG bilayers. In our Martini 2.2 simulations with POPC bilayers, we observe transmembrane cluster formation at *P*/*L* = 0.004 for melittin. The lower threshold in our simulations may reflect differences in lipid composition or simulation protocols. Sun et al. found that T-pores, consisting of transmembrane peptides, have lower energy and higher permeability than U-pores, although T-pores occur less frequently due to kinetic limitations.^23^ This observation aligns with our finding that melittin forms transmembrane clusters more readily than the shorter Aβ(29–42) peptide, likely due to melittin’s ability to span the full bilayer thickness. Furthermore, Su et al. demonstrated that antimicrobial peptides such as Magainin-2 form toroidal pores at high concentrations in liquid-disordered membrane phases, with pore formation being concentration-dependent.^47^ These studies support our observation that peptide concentration plays a crucial role in determining whether peptides remain surface-bound or form transmembrane structures, and that the mechanism of membrane disruption through pore or cluster formation is a common feature among antimicrobial peptides.

### 4.3 Martini 2.2 Better Represents Peptide-Lipid Interactions than Martini 3.0

We found that the Martini 2.2 and Martini 3.0 force fields show quite different peptide-lipid interactions for our systems. For instance, in the Martini 3.0 simulations we did not see transmembrane clusters that were present in the Martini 2.2 simulations, see Figures 8 and 9.

**Figure 8:**
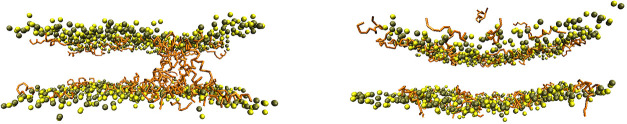
Illustrative example of Aβ(29–42)/POPC interactions in the Martini 2.2 (left panel) and Martini 3.0 (right panel) force fields. Left panel: Martini 2.2 allows for peptide aggregation and insertion into the membrane. Right panel: Martini 3.0 model does not show peptide insertion. Peptides remain at the membrane surface with minimal penetration into the bilayer. Aβ(29–42) residues are shown in orange, PO4 beads in yellow and NC3 beads in moss green.

**Figure 9:**
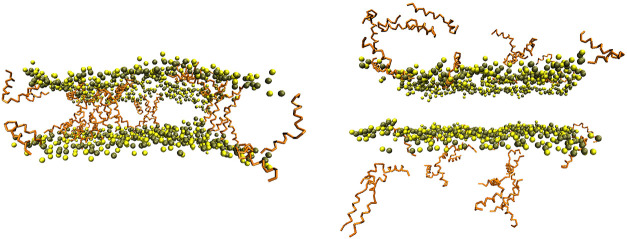
Illustrative example of melittin/POPC interactions in the Martini 2.2 (left panel) and Martini 3.0 (right panel) force fields. Left panel: Martini 2.2 allows for peptide aggregation and insertion into the membrane. Right panel: Martini 3.0 model does not show peptide insertion. Peptides remain at the membrane surface with minimal penetration into the bilayer. Melittin residues are shown in orange, PO4 beads in yellow and NC3 beads in moss green.

The comparison between Martini 2.2 and Martini 3.0 force fields reveals differences in their representation of peptide-lipid interactions. The Martini 3.0 force field appears to generate weaker interactions between peptides and lipid bilayers compared to Martini 2.2. This is particularly evident in the melittin/POPC systems, see Figure 9, where Martini 3.0 simulations showed minimal peptide-membrane interactions, with peptides remaining largely in solution rather than adsorbing to or inserting into the membrane.

The weaker interactions in Martini 3.0 may be attributed to the refined parametrization that was designed to address limitations in Martini 2.2. However, in the context of membrane-active peptides, this refinement appears to have overcorrected the interactions, resulting in systems where peptides do not effectively interact with the membrane.^48^ Recent studies have shown that rescaling protein–water interactions or applying water-bias corrections can improve peptide-membrane binding in Martini 3.^49–51^ However, in this work we chose to use the standard, unmodified versions of both Martini 2.2 and Martini 3.0 force fields to enable a direct comparison between the two force field generations. Applying corrections to Martini 3 while using standard Martini 2.2 would complicate this comparison. This suggests that while Martini 3.0 may provide more accurate representations for certain systems, it may not be optimal for studying peptide-membrane interactions that lead to membrane disruption without additional corrections.

The lack of transmembrane cluster formation in Martini 3.0 simulations, combined with the minimal surface adsorption observed for melittin, indicates that this force field may not be suitable for studying the mechanisms of membrane-active peptides. In contrast, Martini 2.2 appears to better represent the peptide-lipid interactions that can lead to the loss of membrane integrity, making it more appropriate for studying antimicrobial and amyloidogenic peptide effects on lipid bilayers.

## 5 Conclusions

In this work we tested the assumptions and predictions of Huang’s two-state model,^14,15^ using coarse-grained molecular dynamics simulations of an antimicrobial peptide melittin and amyloid peptide fragment Aβ(29–42), interacting with POPC bilayer. In Huang’s model and in our simulations, peptides first adsorb at the membrane interface and then, beyond a critical peptide-to-lipid ratio, insert into the membrane to form transmembrane clusters. However, unlike in Huang’s model, this cooperative effect does not coincide with the turnover in the rate of membrane thinning with increased peptide-to-lipid ratio. Moreover, the assumptions of Huang’s free energy model are not met in our simulations, as both the compression modulus, *K*_a_ and the membrane hydrophobic region volumes, *V*_L_, strongly depend of the peptide-to-lipid ratio. We conclude that Huang’s free energy model cannot rationalize our simulations.

We found that antimicrobial melittin and amyloidogenic Aβ(29–42) show similar characteristics for peptide-lipid interaction. This supports the conjecture that antimicrobial peptides and amyloidogenic peptides may use the same molecular mechanism to disrupt lipid membranes.

In our simulations, the Martini 3.0 force field showed weaker lipid-peptide interactions than Martini 2.2. In our case, Martini 2.2 seems to better represent the peptide-lipid interactions that can lead to the loss of membrane integrity.

## Supplementary Material



## Notes

System-specific details of the simulation setup and numerical values for representative properties of the simulated systems (PDF)
