# Supplementary material for: Concentration Effects in Peptide–Lipid Bilayer Interactions: A Coarse-Grained Molecular Dynamics Study
Source: J Phys Chem B. 2026 Mar 16;130(12):3332–41. doi: 10.1021/acs.jpcb.5c07780 (PMC13362193; doi:10.1021/acs.jpcb.5c07780)
Supplement: Supplementary file 1 [file jp5c07780_si_001.pdf]

Supporting Information for

Concentration Effects in Peptide-Lipid Bilayer Interactions: A Coarse-Grained Molecular Dynamics Study

Aleksandra Drajkowska,* Andrzej Molski†

Adam Mickiewicz University in Poznań, Faculty of Chemistry,

ul. Uniwersytetu Poznańskiego 8, 61-614 Poznań, Poland

January 29, 2026

*adrajkowska@op.pl, corresponding author

†amolski@amu.edu.pl

1 Simulation setup

Aβ(29-42)/POPC, Martini 2.2, Martini 3.0								
P	L	P/L	W	Na^+	Cl^-	Ini. box (nm^3)	Final box (nm^3)	
							Martini 2.2	Martini 3.0
4	512	0.008	11390	305	305	15 × 15 × 15	13.1 × 13.1 × 12.3	13.1 × 13.1 × 11.9
8		0.016					13.1 × 13.1 × 12.2	13 × 13 × 12
16		0.031					13.2 × 13.2 × 12.1	13.1 × 13.1 × 12.3
24		0.047					13.4 × 13.4 × 11.9	13.4 × 13.4 × 12.5
32		0.063					13.4 × 13.4 × 11.9	13.3 × 13.3 × 11.6
48		0.093					13.8 × 13.8 × 11.3	13.7 × 13.7 × 11.1
56		0.111					14 × 14 × 11.2	–
64		0.125					13.9 × 13.9 × 11.2	13.9 × 13.9 × 10.9
66		0.128					14.1 × 14.1 × 11	–
68		0.133					14.1 × 14.1 × 11	–
70		0.137					14.1 × 14.1 × 11	–
72		0.141					14.1 × 14.1 × 11	–
64		0.145					14.2 × 14.2 × 11	–

Table S1: Summary of the Martini 2.2 and Martini 3.0 simulations of Aβ(1-29) in POPC. The concentration of NaCl was 15 mM.

Melittin/POPC, Martini 2.2, Martini 3.0								
P	L	P/L	W	Na^+	Cl^-	Ini. box (nm^3)	Final box (nm^3) Martini 2.2	Final box (nm^3) Martini 3.0
2	512	0.004	11380	305	315	$15 \times 15 \times 15$	$13.2 \times 13.2 \times 12.1$	$13.1 \times 13.1 \times 11.8$
4		0.078	11370		325		$13.0 \times 13.0 \times 12.5$	$13.0 \times 13.0 \times 11.9$
8		0.016	11350		345		$13.2 \times 13.2 \times 12.1$	$13.0 \times 13.0 \times 12.0$
16		0.031	11310		385		$13.5 \times 13.5 \times 11.9$	$13.0 \times 13.0 \times 12.1$
24		0.047	11270		425		$13.9 \times 13.9 \times 11.2$	$13.0 \times 13.0 \times 12.3$
26		0.050	11260		435		$13.7 \times 13.7 \times 11.7$	–
28		0.056	11250		445		$14 \times 14 \times 11.1$	–
30		0.059	11240		455		$14 \times 14 \times 11.2$	–
32		0.063	11230		465		$14.1 \times 14.1 \times 11.0$	$13.3 \times 13.3 \times 11.8$

Table S2: Summary of the Martini 2.2 and Martini 3.0 simulations of Melittin in POPC. The concentration of NaCl was 15 mM.

2 Simulation results

Aβ(29-42)/POPC, Martini 2.2					
P/L	ϕ	h (nm)	A_L (nm^2)	$h \times A_L$ (nm^3)	K_a (pN/nm)
0.008	0	3.87	0.666	2.58	255
0.016	0	3.87	0.668	2.59	250
0.031	0	3.85	0.681	2.62	249
0.047	0	3.82	0.693	2.65	245
0.063*	0.19	3.80	0.709	2.70	247
0.093*	0.77	3.76	0.740	2.78	236
0.111*	0.28	3.73	0.755	2.82	223
0.125*	0.51	3.73	0.767	2.86	208
0.128*	0.42	3.72	0.773	2.88	226
0.133*	0.33	3.73	0.776	2.89	205
0.137*	0.44	3.73	0.778	2.90	203
0.141*	0.49	3.72	0.782	2.91	212
0.145*	0.59	3.75	0.785	2.94	211

Table S3: Fraction of inserted peptides, ϕ, membrane thickness, h, area per lipid, A_L, and stretch modulus, K_a, for POPC bilayer with bound Aβ(29-42) at various P/L ratios. The stars at P/L values indicate the formation of transmembrane clusters. Simulations conducted in Martini 2.2.

Melittin/POPC, Martini 2.2					
P/L	ϕ	h (nm)	A_L (nm^2)	$h \times A_L$ (nm^3)	K_a (pN/nm)
0.004*	0.67	3.88	0.665	2.58	254
0.078*	0.92	3.87	0.674	2.61	250
0.016*	0.92	3.85	0.690	2.65	252
0.031*	0.77	3.82	0.715	2.73	238
0.047*	0.74	3.77	0.745	2.81	214
0.050*	0.82	3.78	0.753	2.85	230
0.056*	0.74	3.75	0.759	2.85	201
0.059*	0.76	3.77	0.761	2.87	184
0.063*	0.92	3.74	0.775	2.90	178

Table S4: Fraction of inserted peptides, ϕ, membrane thickness, h, area per lipid, A_L, and stretch modulus, K_a, for POPC bilayer with bound melittin at various P/L ratios. The stars at P/L values indicate the formation of transmembrane clusters. Simulations conducted in Martini 2.2.

Aβ(29-42)/POPC, Martini 3.0					
P/L	ϕ	h (nm)	A_L (nm^2)	$h \times A_L$ (nm^3)	K_a (pN/nm)
0.008	0	3.85	0.664	2.56	208
0.016	0	3.83	0.670	2.57	207
0.031	0	3.78	0.687	2.60	197
0.047	0	3.76	0.694	2.61	185
0.063	0	3.72	0.708	2.63	174
0.093	0	3.65	0.738	2.69	155
0.125	0	3.58	0.764	2.74	140

Table S5: Fraction of inserted peptides, ϕ, membrane thickness, h, area per lipid, A_L, and stretch modulus, K_a, for POPC bilayer with bound Aβ(29-42) at various P/L ratios. Simulations conducted in Martini 3.0.

Melittin/POPC, Martini 3.0					
P/L	ϕ	h (nm)	A_L (nm^2)	$h \times A_L$ (nm^3)	K_a (pN/nm)
0.004	0	3.81	0.671	2.56	207
0.008	0	3.85	0.662	2.55	198
0.016	0	3.82	0.668	2.55	178
0.031	0.04	3.78	0.681	2.57	150
0.050	0	3.81	0.670	2.55	129
0.063	0.01	3.77	0.679	2.56	149

Table S6: Fraction of inserted peptides, ϕ, membrane thickness, h, area per lipid, A_L, and stretch modulus, K_a, for POPC bilayer with bound melittin at various P/L ratios. Simulations conducted in Martini 3.0.
